# The Silly Season Again

**Published:** 1911-09-09

**Authors:** 


### The Hospital

A JOURNAL OF

The Medical Sciences and Hospital Administration.

NEW SERIES. No. 241, VOL. IX. [No. 1313, VOL. L.] SATURDAY, SEPTEMBER 9, 1911.

### THE SILLY SEASON AGAIN.

At this time of year, as everybody knows, the newspapers are somewhat hard pressed to eke out their columns with copy that shall catch the attention of the British holiday-maker. Genuine news is scarce, and the columns have to be filled somehow. In olden days sea-serpents, Armada galleons, and other prodigies, were regularly resuscitated every August; but even their long innings has at last been declared closed, and nowadays, as often as not, it is some miraculous cure or some newly discovered disease which furnishes the material for "snappy" paragraphs. It is a time of year, in fact, at which any medical man with ambitions to obtain free advertisement in the lay press can achieve them fairly easily; and further, when even those who have no desire for these meretricious distinctions may have to be very wary to escape them.

The most recent example of this tendency to anticipate the proper scientific duly pondered verdict on any given question which is being submitted to research, is to be found in the fuss which is being made over the treatment of various diseases by injections of sea water. To judge by the daily papers, this treatment might have been invented last week; though as a matter of fact it has been under investigation for at least seven years in France, and full accounts of it were long ago published in England. It is with no intention of implying any reflection on those who are now experimenting with this treatment in a dispensary in Soho that we feel constrained to protest against the exploitation of this remedy in the press. To investigate carefully the large claims which have been made in France about the value of sea-water injections is a praiseworthy object, so long as it is done scientifically and honestly. To open a clinic in a poor part of London for free treatment is both excellent in itself, and most timely, in view of the hot summer we have had, and the consequent prevalence of gastro-enteritis among the infant population. But to allow reporters to manufacture sensational paragraphs on the miraculous cures wrought by this new system of therapy, and to "boom" right and left a treatment on which certainly no one has any right as yet to pronounce a mature opinion, this is neither praiseworthy nor even judicious. If the sea-water injection treatment justifies the claims made for it as a beneficial innovation worthy of adoption for gastro-intestinal and cutaneous diseases, it will soon gain adherents, and take its place among the recognised resources of the physician. If it does not, no amount of journalistic clap-trap will avail it in the long run. Meanwhile, if there is one thing more likely than another to make the medical profession in Britain suspicious both of the treatment and of its exponents, it is pernicious indiscriminate laudation in the columns of the daily papers.

As to the treatment itself, those who desire to read for themselves how it is administered will find a comprehensive account of it in the *Medical Annual* for 1910, by Dr. Robert-Simon, of Paris. The idea on which it is based is, to our minds, frankly nonsensical: a M. Quinton holds that animal life first appeared in the sea, and therefore that sea water is of the utmost value to animal life now. Our astonishment at this most illogical proposition increases to amazement when we read of its further development. Thus the sea contains 3.3 per cent. of salts, we are told; but Quinton holds that the primordial ocean in which life first began contained but 0.8 per cent. He concludes hence that a 0.8 per cent. solution of the salts dissolved in sea water represents the medium for "optimum cellular activity," and this is gravely enunciated as the "law' of marine constancy"! How the conclusion as to the composition of the primordial ocean is reached Dr. Robert-Simon does not explain; nor is it apparent why it should be concluded that the salts which have been added to the ocean since those prehistoric times are the same and in the same proportions as the 0.8 per cent. already existing there. But, indeed, there are other passages in Dr. Robert-Simon's argument where the reasoning is as faulty as in the examples just quoted.

Founded, therefore, on a hypothesis which can only be described as laughable, the practical workings of the system require the closest scrutiny. It may, of course, in practice fulfil all that is claimed for it, although the underlying hypothesis is merely ridiculous. Certainly Dr. Robert-Simon produces some remarkable cases in which obstinate and dangerous diseases have responded rapidly to the sea-water treatment; and he admits failures in a very open and candid way. The conditions for which he recommends the treatment are gastro-enteritis of infants and adults; anæmia and chlorosis; eczema, psoriasis, and ulcers of the skin; syphilis and surgical tuberculosis; infantile paralysis, chorea, and exophthalmic goitre: a formidable list! To be just, he makes out a case for investigation; but that investigation, to have any value, must be properly conducted on scientific principles, otherwise a door is opened for all sorts of quackery. And, finally, the results must be communicated to the medical, not the lay, press, if they are to command any confidence at all from the medical profession. Already a number of papers on the results obtained by Continental and American investigators have been published, and the conclusions drawn from such research are by no means unanimously in favour of the new method of treatment.

